# Efficacy and Tolerability of a New Formulation in Rectal Ointment Based on Zn-L-Carnosine (Proctilor®) in the Treatment of Haemorrhoidal Disease

**DOI:** 10.3389/fsurg.2022.818887

**Published:** 2022-03-24

**Authors:** Renato Pietroletti, Antonio Giuliani, Alberto Buonanno, Antonella Mattei, Fabiana Fiasca, Gaetano Gallo

**Affiliations:** ^1^Surgical Coloproctology University of L'Aquila—Hospital Val Vibrata, Sant'Omero, Italy; ^2^General Surgery University of L'Aquila—Hospital San Salvatore, L'Aquila, Italy; ^3^General Surgery ASREM-AREA 5, Hospital San Benedetto del Tronto, San Benedetto del Tronto, Italy; ^4^Public Health Section—Department of Life Health and Environmental Sciences University of L'Aquila, L'Aquila, Italy; ^5^Department of Surgery University “Magna Graecia” of Catanzaro, Catanzaro, Italy

**Keywords:** haemorrhoids, rectal bleeding, thrombosed piles, topical treatment, Zinc-L-Carnosine

## Abstract

Haemorrhoidal disease (HD) shows high prevalence in western countries, reaching 4.4% per year in the US. Topical preparations are the first-line treatments, which are readily available as “over-the-counter” (OTC) products, often containing a nonstandardised mixture of “natural” remedies, or anaesthetics or cortisol;those latter are not free from undesirable effects. The Zinc-L-Carnosine is a cytoprotective compound, promoting mucosal repair in the gastrointestinal tract and also in mucosal repair, following radiation injuries to the rectum as well as in ulcerative colitis. Our aim was to study the efficacy of Zinc-L-Carnosine in relieving acute symptoms of HD, testing a preparation in the rectal ointment, Proctilor®, in patients complaining of bleeding or thrombosed piles. In a multicentre open trial, 21 patients older than 18 years of age were enrolled. The symptoms of HD were graded according to the Haemorrhoidal Disease Symptoms Score (HDSS) in association with the Short Health Scale (SHS) to assess the influence of HD on quality of life. The pain was assessed with the VAS score, bowel habit by means of the Bristol scale. The patients were evaluated at enrolment (T0) and 2 (T1) and 4 (T2) weeks of treatment with Proctilor® rectal ointment. There were 10 men and 11 women; mean age, 49 years. Pain, bleeding, and thrombosis were all significantly reduced after treatment; the mean VAS score decreased from 4.71 ± 3.05 at T0 to.52 ± 0.87 and.05 ± 0.22 at T1 and T2, respectively; (mean ± SD; *p* < 0.001 in both cases). Similarly, the HDSS score showed to be significantly reduced between T0, T1 (8.05 ± 4.55 vs. 1.14 ± 1.01), and T2 (8.05 ± 4.55 vs. 24 ± 0.44) (mean ± SD; *p* < 0.001 in both cases). Quality of life showed to be improved as the SHS score decreased significantly with treatment (7.90 ± 4.17 at T0 vs. 4.24 ± 0.44 at T1 vs. 4.05 ± 0.22 at T2; mean ± SD; *p* < 0.001 in both cases). The Bristol score of defecation remained substantially unchanged. No side effects or discontinuation of treatment were reported. Results of our investigation suggest a role of Proctilor® rectal ointment in treating symptomatic HD with good results and an excellent safety profile. However, our preliminary results encourage further studies on a larger number of patients to confirm the role of Zinc-L-Carnosine in the rectal ointment for the topical treatment of HD.

## Introduction

Haemorrhoidal disease (HD) has a high prevalence (1); sedentary life, alimentary habits, constipation or diarrhoea, pregnancy, and physical efforts or straining at defecation may give rise to various clinical features of the haemorrhoidal disease, such as bleeding, prolapse, tenesmus, and anal pain (2). These symptoms may be severe, disabling, and can cause significant worsening of the patient's quality of life, therefore, requiring particular attention. Prevalence of about 4.4% per year has been estimated in the US population, with a peak between 45 and 65 years of age (1). The most common treatments are based upon preparations for topical use, at least in the early phase, and they are generally appreciated by the patient because of the prompt soothing effect.

So far, the topical treatment of HD is not standardised and is based on common anti-inflammatory remedies represented by ointments, enemas, or suppositories containing cortisone or mesalazine and local anaesthetics (3). It is well-known, however, that a prolonged and/or repeated local use of these products is not free from undesirable effects. In addition, the commonly available topical treatments in HD did not show high efficacy both in short-term and long-term remission of symptoms (3).

Hepilor® is an available compound based on Zinc-L-Carnosine, which has shown a significant ability to protect mucous membranes and promote tissue repair. It was extensively tested in the gastro-oesophageal and intestinal areas, without absorption (4–11).

This compound proved to be very effective in treating radiation proctitis, a difficult clinical condition, significantly improving endoscopic pictures and symptoms (12, 13). Furthermore, the product has been effectively tested in ulcerative colitis, where it induced a significant clinical and endoscopic remission (14).

Last but not least, an oral Zn-L-carnosine prevented oesophageal injuries and severe dysphagia in patients with breast cancer who are undergoing radiotherapy (15), resulting in reduced intake of steroids and analgesics.

In summary, the various actions of Zn-L-Carnosine, such as high muco-adhesiveness, increase in mucosal resistance and mucous production, injury repair, and antioxidant radical scavenger action (3–6), are all focused on tissue repair. Thus, based on this background, the compound may exert therapeutic action in HD, counteracting inflammatory changes and tissue damage, and especially relieving acute symptoms.

## Materials and Methods

This was a multicentre open-label uncontrolled trial. The criteria of the STROBE checklist (16) were fulfilled. Adult patients (>18 years old) with symptomatic HD were enrolled. Written informed consent was obtained from all the patients. Exclusion criteria were: age <18; pregnancy; inflammatory bowel disease; viral, bacterial, or radiation proctitis; rectal or anal cancer; anal condylomata; proven or suspected intolerance or hypersensitivity to Zn-L-Carnosine or one or more excipients; and inability to return for a control visit.

Demographic data, symptoms of HD, and details of the enrolled patients were collected in a dedicated database.

Enrolled patients underwent clinical examination and anoscopy to assess the degree of HD according to the Goligher classification and to detect any active bleeding. The presence of thrombosis was registered and was defined as a bulging, swollen perianal vein that is pinkish or bluish in colour. Stool characteristics were assessed using the original version of the Bristol Stool Form Scale (17); severity of symptoms was quantified using the “haemorrhoidal disease symptom score” (HDSS) (18), in which all symptoms were graded with a 5-item scale (0 = never, 1 = <1 time a month, 2 = <1 time a week, 3 = 1–6 days a week, and 4 = every day or always; Minimum = 0, Maximum = 20) in association with “short health scale” (SHS) to assess the burden of HD on quality of life with four questions using a 7-point Likert Scale (minimum = 4, maximum = 28). The pain was recorded using a visual analogue scale (VAS) (the minimum score = 0, maximum = 10). These parameters were recorded at the time of the enrolment (T0) with anthropometric characteristics of the patients and follow-up visits after 2 weeks (T1) and after 4 weeks (T2).

All the patients underwent treatment with the application of Proctilor® rectal ointment 3 ml three times a day. The product was directly provided by the manufacturing company to the Experimentation Centres.

### Statistical Analysis

The demographic and clinical characteristics of the sample were analysed using descriptive statistics. The discrete and nominal variables were expressed using frequencies and percentages; continuous variables were expressed as mean values with relative standard deviations (± SDs).

The ANOVA for repeated-measures (RM) was used to determine the significance of the difference in scores (VAS, HDSS, Bristol, and SHS) for the time trend after a logarithmic transformation for the normalisation of the data as the non-normal distribution verified through the Shapiro–Wilk test. Logarithmic transformation of the scores over time was represented graphically. *Post-hoc* analysis was performed using Bonferroni's method for pairwise comparisons when the resulting differences were statistically significant. The differences in the clinical characteristics of the sample (bleeding and thrombosis) were analysed using Cochran's *Q*-test for the time trend. *Post-hoc* analysis was performed using McNemar's test. The power of the study was evaluated using the F test, comparing mean values of VAS at t0 (4.7), t1 (0.5), and t2 (0.04). The estimated power (1–β) was 94%. A prospective analysis with 94% of power and a level of significance α = 0.05 identified an estimate of the sample size for a crossover design of at least 21 participants. The data were recorded electronically, and statistical analyses were carried out using Stata Statistical Software, Release 15 (Stata Corp LP, College Station, TX, U.S.A.). All the tests were 2 tailed, and *p* < 0.05 were considered statistically significant.

## Results

A total of 21 patients met the criteria to be included in this study. There were 10 men (47.6%) and 11 women (52.4%); the mean age was 49 years (range, 25–74). All of them were diagnosed with symptomatic HD. The f was diagnosed in nine patients (42.9%). In Table 1, we reported the demographic characteristics of the sample.

**Table 1:** Characteristics of the patients enrolled.

	**Total**
	***N* = 21**
**Sex**, ***n*** **(%)**	
Female	10 (47.62)
Male	11 (52.38)
Age, mean ± SD	49.38 ± 14.86
**Fissures**, ***n*** **(%)**	
No	12 (57.14)
Yes	9 (42.86)
**Bleeding**, ***n*** **(%)**	
No	6 (28.57)
Yes	15 (71.43)
**Thrombosis**, ***n*** **(%)**	
No	9 (42.86)
Yes	12 (57.14)

**Figure 1:**
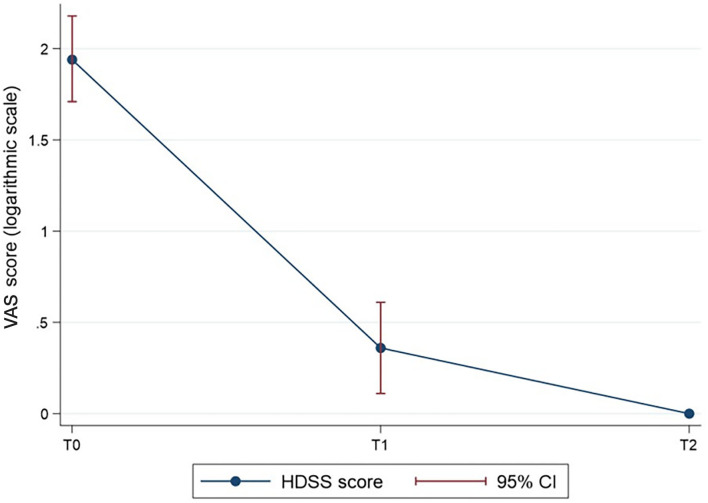
The HDSS score over time (log-transformed for normalisation of the VAS score data). *P* < 0.001 for T1 vs. T0 and T2 vs. T0.

When stratified for time is of observation, the reduction of the VAS score (Table 2) was statistically significant (*p* = 0.018). The VAS score over time is shown in Figure 1, with logarithmic transformation for the normalisation of the data. The results of the *post-hoc* analysis (Table 3) indicated statistically significant differences in the VAS score between time 0 and 1 (4.71 ± 3.05 vs. 52 ± 0.87), and time 0 and 2 (4.71 ± 3.05 vs. 05 ± 0.22) (*p* < 0.001 in both cases).

**Table 2:** Scores values, stratified for time of observation.

	**T0**	**T1**	**T2**	*p*-value^*^
VAS, mean ± SD	4.71 ± 3.05	0.52 ± 0.87	0.05 ± 0.22	**0.018**
HDSS, mean ± SD	8.05 ± 4.55	1.14 ± 1.01	0.24 ± 0.44	**<0.001**
Bristol, mean ± SD	2.90 ± 1.67	3.29 ± 0.78	3.43 ± 0.60	0.112
SHSHD, mean ± SD	7.90 ± 4.17	4.24 ± 0.44	4.05 ± 0.22	**0.001**

* *One-way repeated measures ANOVA*.

*Bold values denote statistical significance*.

**Table 3:** *Post-hoc* pairwise comparisons of the scores using Bonferroni's method.

	**T0 vs. T1**	**T0 vs. T2**	**T1 vs. T2**
VAS	**<0.001**	**<0.001**	0.999
HDSS	**<0.001**	**<0.001**	0.485
SHSHD	**<0.001**	**<0.001**	0.999

*Bold values denote statistical significance*.

**Figure 2:**
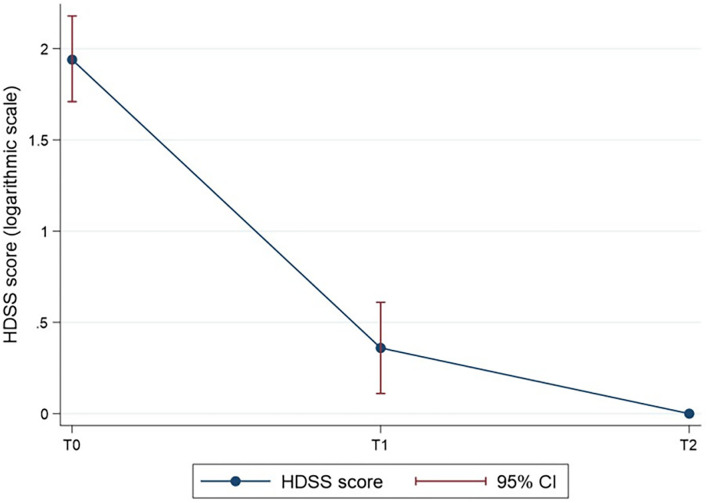
The HDSS score over time (log-transformed for normalisation of the HDSS score data). *P* < 0.001 for T1 vs. T0 and T2 vs. T0.

The difference in the mean HDSS score (Table 2) was statistically significant over time (*p* < 0.001). The HDSS score at a given time is shown in Figure 2, with logarithmic transformation for the normalisation of the data. The results of the *post-hoc* analysis (Table 3) indicated statistically significant differences of the HDSS score between time 0 and 1 (8.05 ± 4.55 vs. 1.14 ± 1.01), and time 0 and 2 (8.05 ± 4.55 vs. 24 ± 0.44) of observation (*p* < 0.001 in both cases).

The difference in the mean Bristol score (Table 2) was not statistically significant over time.

**Figure 3:**
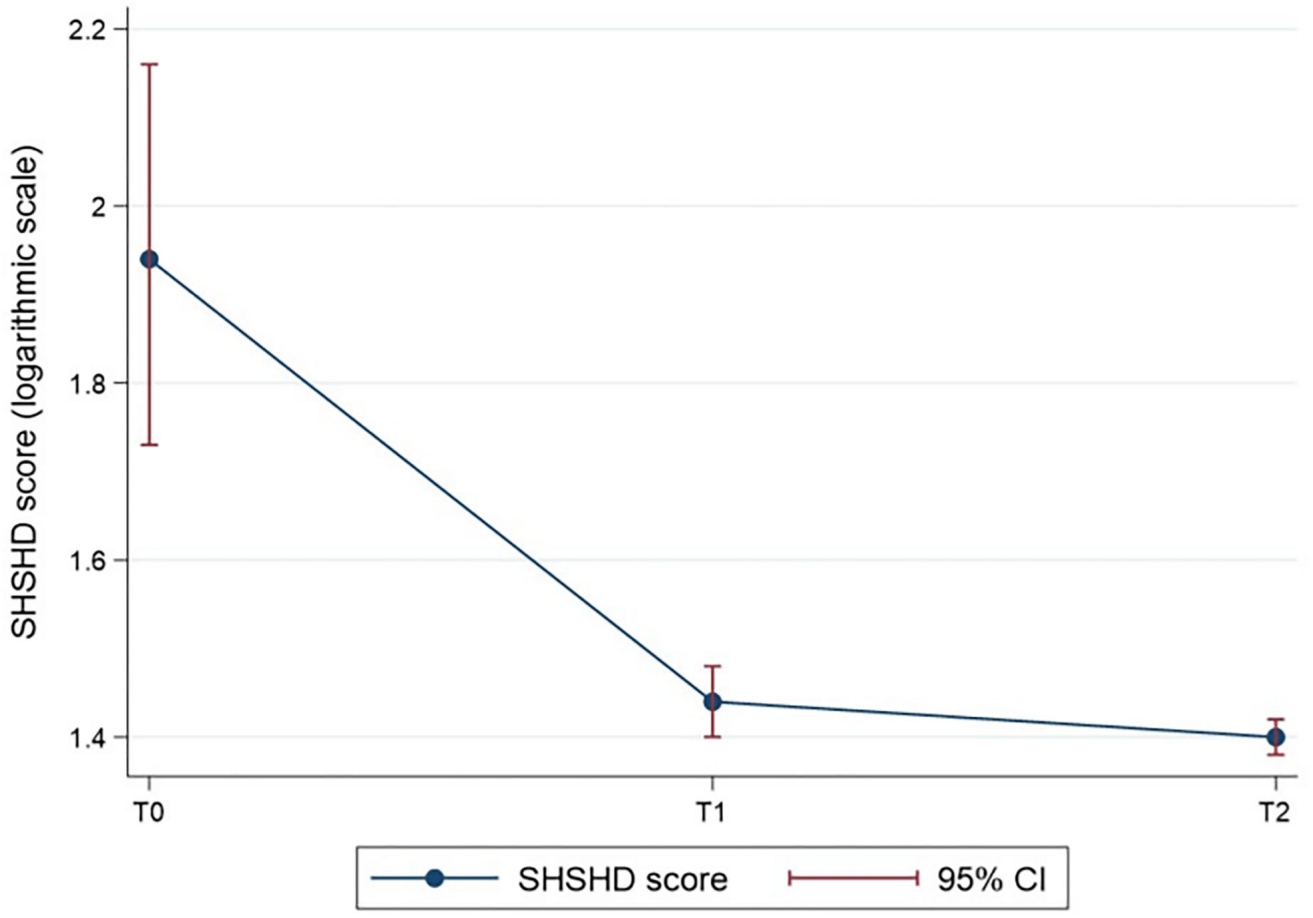
The SHSHD score over time (log-transformed for normalisation of the SHSHD score data). *P* < 0.001 for T1 vs. T0 and T2 vs. T0.

The difference in the mean of the Short Health Scale Haemorrhoidal Disease (SHSHD) score (Table 2) was statistically significant over time (*p* = 0.001). The SHSHD score at a given time is shown in Figure 3, with a logarithmic transformation for the normalisation of the data. The results of the *post-hoc* (Table 3) analysis indicated statistically significant differences of the SHSHD score between time 0 and 1 (7.90 ± 4.17 vs. 4.24 ± 0.44), and time 0 and 2 (7.90 ± 4.17 vs. 4.05 ± 0.22) of observation (*p* < 0.001 in both cases).

Significative differences also emerged for clinical characteristics (bleeding and thrombosis) (Table 4). Cochran's *Q*-test showed significant different frequencies of bleeding (*p* = 0.005) and thrombosis (*p* < 0.001) at the various times of observation, with higher percentages of bleeding (71.43 vs. 9.52%) and thrombosis (57.14 vs. 9.52%) at time 0 respect to time 2. The results of the *post-hoc* (Table 5) analysis indicated statistically significant differences between time 0 and 1, and time 0 and 2 of observation (*p* = 0.002 and *p* < 0.001, respectively) for bleeding.

**Table 4:** Clinical characteristics of the sample, stratified for time of observation.

	**T0**	**T1**	**T2**	*p*-value^*^
**Bleeding**, ***n*** **(%)**				**0.005**
No	6 (28.57)	16 (76.19)	19 (90.48)	
Yes	15 (71.43)	5 (23.81)	2 (9.52)	
**Thrombosis**, ***n*** **(%)**				**<0.001**
No	9 (42.86)	19 (90.48)	19 (90.48)	
Yes	12 (57.14)	2 (9.52)	2 (9.52)	

* *Cochran's Q-test*.

*Bold values denote statistical significance*.

**Table 5:** *Post-hoc* pairwise comparisons clinical characteristics of the sample using McNemar's test.

	**T0 vs. T1**	**T0 vs. T2**	**T1 vs. T2**
Bleeding	**0.002**	**<0.001**	0.257

*Bold values denote statistical significance*.

## Discussion

HD is a common complaint affecting ~40–50% of the adult population (19).

Its pathophysiology has been clarified, and it is characterised by a degenerative process of collagen and fibroelastic supportive fibres of the vascular cushions, resulting in abnormal dilatation, weakness, and displacement of the cushions, resulting in prolapse, bleeding, and thrombosis (20), although many aspects remain controversial (21). Conservative treatments are the first-line options for all degrees of haemorrhoidal disease (3). Conservative treatments are first line options for all degrees of HD (3, 22), and are really helpful in figuring out which patient needs a personalized treatment (23, 24).

A compound based on Zinc-L-Carnosine has shown a significant ability to protect mucous membranes and to promote tissue repair. Zinc is a mineral with many important functions, including the healing of damaged tissue. It seems that insulin-like growth factor-I (IGF-I) could be one of the main factors involved in the healing effect of zinc on gastric ulcers (25).

Hepilor® has been used for the treatment of peptic ulcers in Japan since 1994, and many studies tested it in the gastro-oesophageal and intestinal areas (4–11). In the anorectal field, this compound has proven to be very effective in treating radiation proctitis (12, 13) and ulcerative colitis, where it induced a significant clinical and endoscopic remission (14). Zinc-L-Carnosine induces mucosal repair, stimulating mucus production, antioxidant activity, and membrane-stabilising activity. Its effects are related to different cytoprotective mechanisms, such as the suppression of lipid peroxidation, reduction of the levels of cytokines, and inhibition of superoxide generation. In this way, this compound seems to promote the restoration of the mucosa (25).

Therefore, based on its characteristics and the good results obtained from the use of Zinc-L-Carnosine in tissue repair on the rectal mucosa, it may be possible to hypothesise an effective therapeutic use in the context of other anorectal pathologies. Among these, HD, with its acute or chronic complications, is characterised by a large epidemiological, social, and economic impact.

Although with the limitations due to the small sample of patients, our preliminary study demonstrated the effectiveness of the Zinc-L-Carnosine rectal cream (Proctilor®) in relieving common symptoms of HD, such as bleeding or thrombosis. In fact, we observed a significant reduction in the intensity of the pain at the VAS score, as well as in HDSS and SHSHD scores in our patients between the time of diagnosis and the last day of treatment.

Constipation and bowel habit are believed to play a role in the development of HD (1–3), and the improvement of bowel habit has a documented therapeutic effect on initial symptoms of HD. The bowel function of our patients ranged from normal to mild constipation and improved after giving pieces of dietary advice. Interestingly, the Bristol stool scale score did not show statistically significant differences between T0 (the mean score, 2.90) and T1 and T2 (mean scores 3.29 and 3.43, respectively). These results further support the role of Proctilor® in treating HD, given the low impact of constipation treatment in our patients as demonstrated by the substantially normal score of the Bristol stool scale.

As for the clinical complaints reported by our patients, the main goal of treatment was to obtain a prompt relief of their symptoms. This goal was reached since significant differences emerged in reduction of pain, improvement of HDSS, and improvement of quality of life (SHSHD), which is already at T1 after 2 weeks of treatment. In addition, a statistically significant improvement was also reported for bleeding and thrombosis between T0–T1 and T0–T2. Zn-L-carnosine rectal ointment seems to show a fast therapeutic effect, as requested commonly by the patients affected by HD, possibly avoiding oral or parenteral administration of substances.

This product showed an excellent safety profile since no patient complained of side effects nor discontinuation of treatment was needed for adverse events. This is important since many topical agents for treatment of HD, containing local anaesthetics or cortisol, are prone to develop local discomfort in chronic use. Four weeks of treatment are quite a long period of topical administration, reasonably sufficient to show adverse events in our patients, thus, reassuring about the high tolerability of Proctilor® rectal ointment.

In conclusion, the very preliminary results of our investigation suggest the effectiveness of Proctilor® ointment for the management of low-grade HD in terms of reduction of pain, bleeding, and relief of thrombosis. It is undoubtedly difficult to draw definitive conclusions due to the small number of patients in this retrospective cohort. However, with these limitations, our study poses the premises for further investigations on a larger number of individuals to confirm the potential therapeutic role of Zn-L-Carnosine in the topical treatment of HD.

## Data Availability Statement

The raw data supporting the conclusions of this article will be made available by the authors, without undue reservation.

## Ethics Statement

Ethical review and approval was not required for the study on human participants in accordance with the local legislation and institutional requirements. The patients/participants provided their written informed consent to participate in this study.

## Author Contributions

RP, AG, AB, and GG recruited patients and made the observational study, selecting patients making diagnosis, treatment, follow-up and they collected data, and elaborated the results. AM and FF made statistical analysis. All authors participated in the elaboration of the manuscript and made final approval of the paper.

## Funding

This study was supported by the Company Azienda Farmaceutica Italiana.

## Conflict of Interest

The authors declare that the research was conducted in the absence of any commercial or financial relationships that could be construed as a potential conflict of interest.

## Publisher's Note

All claims expressed in this article are solely those of the authors and do not necessarily represent those of their affiliated organizations, or those of the publisher, the editors and the reviewers. Any product that may be evaluated in this article, or claim that may be made by its manufacturer, is not guaranteed or endorsed by the publisher.

## Abbreviations

HD: Haemorrhoidal disease
VAS: Visual analogue scale
HDSS: Haemorrhoidal disease symptoms score
SHS: Short health scale.
